# Perceived Crisis Management and Post-Traumatic Growth among Chinese Preschool Teachers during COVID-19: Risk Perception as a Moderator

**DOI:** 10.3390/ijerph192013697

**Published:** 2022-10-21

**Authors:** Xiumin Hong, Youpeng Liu, Mingzhu Zhang

**Affiliations:** 1 Faculty of Education, Beijing Normal University, No. 19 Xin Jie Kou Wai Street, Hai Dian District, Beijing 100875, China; 2 Shandong Province Pre-Primary Education Centre, No. 8 Nan Xu Men Wai Street, Li Xia District, Jinan 250012, China

**Keywords:** post-traumatic growth, perceived crisis management, risk perception, preschool teachers, COVID-19, Chinese

## Abstract

This study explored post-traumatic growth among preschool teachers during COVID-19 and investigated associations among post-traumatic growth, perceived crisis management, and risk perception. The participants were 2921 Chinese preschool teachers (96.5% women). Teachers’ reports of post-traumatic growth, perceived crisis management, and risk perception were analyzed by multivariate techniques. The results revealed that preschool teachers’ post-traumatic growth was at an intermediate level, and there was no significant difference in post-traumatic growth by risk level area. Post-traumatic growth was significantly related to risk perception and perceived crisis management, and risk perception appeared to moderate the relationship between perceived crisis management and post-traumatic growth. Our findings highlight the importance of considering the roles of perceived crisis management and risk perception in preschool teachers’ post-traumatic growth. Related suggestions for preschool teachers’ mental health are discussed.

## 1. Introduction

Preschool teachers work with young children and have major responsibilities, including children’s care and safety and teaching organization. They often face high levels of stress in their work, which highlights the emotionally challenging nature of the teaching profession [1,2,3]. A survey of 674 childcare workers in the USA found that 36.1% met or exceeded the criteria for clinical depression [4]. In 37 Head Start programs in Pennsylvania, 24% of teachers had clinically significant depressive symptoms [5]. In China, the level of mental health among preschool teachers was significantly low [6].

The outbreak of COVID-19 caused various psychological changes, such as increased stress, anxiety, panic attacks, and depression [7]. The mental health of preschool teachers was threatened during COVID-19. Many preschools changed their mode of organization and operation, which placed teachers in a difficult position in terms of working conditions. Uncertainty about self-safety, health, working income, and professional identity further increased preschool teachers’ anxiety [8,9,10,11]. Preschool teachers are important participants in children’s lives, and their mental health affects children’s growth [12]. Several scholars have made urgent calls for the collection of mental health data and interventions for mental health risks in the context of COVID-19 [11,13].

Although the negative effects of COVID-19 on preschool teachers were evident around the world, positive psychology suggests that disasters may also trigger positive psychological changes and growth. A positive psychological change experienced by people following traumatic events is defined as post-traumatic growth [14,15]. Some people have reported that they experienced post-traumatic growth during COVID-19 [16,17,18,19]. Preschool teachers who achieved post-traumatic growth may have maintained a positive emotional state in their work during COVID-19. In addition, previous research found that traumatic events with different risk levels (e.g., family issues, relationship problems, and the death of someone close) bring people different degrees of post-traumatic growth [20,21]. This may mean that preschool teachers living in different risk areas achieved different levels of post-traumatic growth in the context of COVID-19. 

During COVID-19, positive actions by governments to fight the pandemic may have alleviated negative emotions, promoted patriotic enthusiasm, and increased people’s trust in society [22,23]. Therefore, government actions may also have meant that preschool teachers perceived more external support, which may have helped their post-traumatic growth during COVID-19 [24,25]. Perceptive factors may play a greater role in post-traumatic growth than external factors and could moderate the relationship between external support and post-traumatic growth [15,26,27]. Preschool teachers’ perceptions of risk may have an advantage in terms of influencing post-traumatic growth among preschool teachers. In particular, risk perception may moderate the relationship between crisis management and post-traumatic growth.

Few studies have analyzed post-traumatic growth among preschool teachers and its relationships with risk perception and crisis management by the government in the context of COVID-19. Research focused on Chinese preschool teachers remains scarce. This study explored post-traumatic growth among preschool teachers in China, including differences in post-traumatic growth among preschool teachers living in areas with differing COVID-19 risk levels. Moreover, we tested the moderating role of risk perception in the association between post-traumatic growth and perceived crisis management.

## 2. Literature Review 

### 2.1. Preschool Teachers’ Mental Health during COVID-19

With the spread of COVID-19 globally, the public were immersed in fear, anxiety, and sadness [28]. Travel restrictions also required people to stay at home for long periods, resulting in increased psychological distress [7]. In China, government departments and social institutions provided lectures and guidelines on psychological crisis intervention for affected people via the Internet to help improve people’s mental health [29].

Preschool teachers’ mental health problems were a concern in the context of COVID-19. For example, some preschools faced economic difficulties and the risk of bankruptcy. Challenges such as these led to falling wages among preschool teachers [10]. Most preschool teachers also experienced dual stress from family and work because of the policy of working from home in China [8]. In addition, the relationships between teachers and children were damaged by the reduced communication during COVID-19, which seriously threatened preschool teachers’ professional identities [11]. Some teachers were worried about children’s safety and health in preschools because of susceptibility to infection. 

In China, preschool teachers provide education and care for children aged 3–6 years whose physical and mental development is immature. Preschool teachers’ mental health has a huge impact on children’s lifelong development [6,30]. Therefore, it is urgent to consider the mental health of preschool teachers during COVID-19. Specifically, we should investigate post-traumatic growth among preschool teachers and related factors, such as risk perception and crisis management.

### 2.2. Post-Traumatic Growth during COVID-19

Post-traumatic growth refers to positive psychological changes experienced by individuals following traumatic events [14,15]. It is reflected in a greater appreciation for life, improved relationships with others, an improved sense of personal strength, recognition of new possibilities, and spiritual development [27]. Previous studies have confirmed that people can acquire this growth after experiencing traumatic events [31,32]. 

With the outbreak of COVID-19, researchers explored post-traumatic growth among people living in affected areas. Stallard et al. (2021) found that most caregivers of children reported experiencing post-traumatic growth, including improved interpersonal relationships, a better appreciation of life, and positive mental changes [16]. Other studies found that medical staff and students achieved different degrees of post-traumatic growth during the fight against COVID-19 [18,19,33,34,35]. These studies confirmed the presence of post-traumatic growth among people during COVID-19. 

Preschool teachers are indispensable in the life of children and paying attention to their post-traumatic growth is particularly meaningful in the context of COVID-19. If teachers experienced post-traumatic growth during COVID-19, their mental state may have changed in a positive direction [15,36]. Unfortunately, compared with the above groups, post-traumatic growth during COVID-19 among preschool teachers has largely been ignored. Therefore, it is necessary to explore the profile of preschool teachers’ post-traumatic growth during COVID-19. 

Furthermore, different threat levels and different traumatic events have differing effects on post-traumatic growth [21,37]. This suggests that preschool teachers living in different risk areas could experienced different post-traumatic growth. 

### 2.3. Perceived Crisis Management and Post-Traumatic Growth 

Crisis management refers to a process of planning, decision-making, and resolution carried out by government departments to deal with various crisis situations, and crisis management is a part of the government’s responsibility [38]. Its purpose is to eliminate or reduce the threats and losses caused by the crisis. Perceived crisis management refers to people’s evaluation toward the government’s performance in crises [22,38]. The public expects the government to take measures to manage a crisis in a timely and effective manner [39]. 

Restrictions on local and international travel and in-house isolation were the most common measures enforced by governments worldwide to contain the virus during the COVID-19 pandemic [40]. In China, the government implemented many effective measures to prevent the transmission of the virus, including suspending public transportation, implementing measures based on the level of COVID-19 risk, and developing guidelines for intervening in a mental health crisis. This highlighted the fact that managing the COVID-19 crisis was perceived as important globally. 

Crisis management is closely related to post-traumatic growth. The public’s assessment of governmental crisis management is a problem of public sentiment and psychology [41,42]. During COVID-19, the government’s positive actions to fight the virus may have alleviated negative emotions and promoted people’s patriotic enthusiasm [22,23]. Previous studies showed that the government’s support had a positive relationship with post-traumatic growth [24]. In the context of COVID-19, the government’s crisis management (as a special type of social support) may have promoted post-traumatic growth. That is, individuals may have felt more security from the government, which helped them to engage in more rumination and thereby experience post-traumatic growth [25,43]. Therefore, perceived crisis management is conducive to preschool teachers’ psychological security as well as their trust in the government. The perception of crisis management therefore deserves attention to improve mental health among preschool teachers. In addition, the specific relationship between crisis management and post-traumatic growth among preschool teachers should be explored.

### 2.4. Risk Perception as a Potential Moderator 

Risk perception refers to people’s assessments about the risks associated with various stimuli based on personal subjective and intuitive judgments [44]. The severity, susceptibility, and response methods for risk are key factors that have received the most attention [44,45]. Risk perception effectively predicts people’s response behavior in the face of emergencies [46,47].

Risk perception is closely related to post-traumatic growth. Traumatic events affect people’s basic cognitive schema and trigger painful emotions; people reflect on these traumatic events repeatedly to alleviate the negative impacts [15]. These perception processes are crucial to forming post-traumatic growth because they help people focus more attention on existential matters and dilute illusionary views that are incompatible with their circumstances after the emergence of a negative event [15]. Therefore, post-traumatic growth reflects a positive change in a cognitive schema, and cognitive processing plays a key role in this process [26,48]. Appropriate risk perception, as an integral part of individual cognition, promotes individuals to think about the traumatic events rationally and reasonably, which facilitates post-traumatic growth [49,50].

In addition, risk perception may moderate the relationship between post-traumatic growth and external factors. Armeli et al. (2001) and Stockton et al. (2011) noted that this growth depends on an individual’s cognition of stress events, and the cognition of traumatic events is the main reason for post-traumatic growth [20,51]. Zhou and colleagues (2014) believed that post-traumatic growth was the result of an individual’s cognitive changes, especially from passive to active rumination [25]. A previous study among college students showed a significant positive correlation between risk perception and post-traumatic growth during COVID-19, which suggested the pandemic affected students’ post-traumatic growth through risk perception [19]. Therefore, we believe that compared with external support, risk perception plays a more critical role in forming post-traumatic growth and may moderate the relationship between post-traumatic growth and external factors. However, few studies have investigated the moderating role of risk perception. Whether risk perception plays a moderating role in the relationship between perceived crisis management and post-traumatic growth in the context of COVID-19 remains to be explored.

### 2.5. The Present Study

Identifying factors that affect post-traumatic growth may be necessary to protect preschool teachers’ mental health and promote their post-traumatic growth [40]. However, few studies have explored post-traumatic growth among preschool teachers and its relationships with risk perception and perceived crisis management. In particular, research focused on Chinese preschool teachers remains scarce. The main goals of the present study were to clarify the basic profile of post-traumatic growth among preschool teachers in China during COVID-19 and examine differences in post-traumatic growth among preschool teachers living in different COVID-19 risk areas.

Moreover, we investigated the moderating role of risk perception in the relationship between perceived crisis management and post-traumatic growth. In terms of the moderating role, we hypothesized that no relationship would be found between post-traumatic growth and perceived crisis management at higher levels of risk perception. In contrast, we expected that there would be a positive relationship between post-traumatic growth and perceived crisis management at a relatively moderate level of risk perception.

Therefore, this study explored the following questions. (a) What is the profile of post-traumatic growth among preschool teachers in China in the context of COVID-19? (b) What are the characteristics of post-traumatic growth among preschool teachers living in different risk areas during COVID-19? (c) What are the relationships among crisis management, post-traumatic growth, and risk perception?

Currently, COVID-19 continues to spread worldwide and affects preschool teachers’ mental health. Teachers’ mental health has a huge impact on children’s development. The post-traumatic growth of preschool teachers has not received attention. Therefore, it is urgent to consider the post-traumatic growth among preschool teachers and related factors. In this context, we believe that this study contributes to confirming the factors affecting post-traumatic growth among preschool teachers, which would help government departments and preschools formulate targeted practical measures to improve teachers’ well-being and minimize mental health risks during COVID-19. To our knowledge, this is the first study focused on post-traumatic growth and related factors among preschool teachers in the context of COVID-19. This study will enrich academic research on preschool teachers’ mental health.

## 3. Method

### 3.1. Participants

To make up a representative sample, three geographical and economical divisions were selected because they contained variability among eastern, central, and western China. Meanwhile, when we selected the provinces in the three divisions, we considered their epidemic risk. Specifically, Shandong and Hainan province in the eastern, Hubei and Jilin in the central, and Xinjiang, Gansu, Guizhou, Qinghai, and Ningxia province in the western regions were selected in this study. Participants in these provinces were randomly recruited from the National Training Program for Preschool Teachers during the COVID-19 epidemic in 2020 in China. All respondents were 2921 preschool teachers, 96.5% were women, 32.1% were aged 29 years or below, 47.4% had finished up to 3 years of college, 40.8% had more than 6 years of work experience, and 66.3% were from public preschools. In addition, 23.1% of preschool teachers lived in low-risk areas (0–199 COVID-19 cases), 41.5% were in medium-risk areas (200–9999 COVID-19 cases), and 35.4% were in high-risk areas (≥10,000 COVID-19 cases) (Table 1).

### 3.2. Measures

Post-Traumatic Growth. The measure used to assess post-traumatic growth was the Chinese version of the Post-Traumatic Growth Inventory (PTGI) [52]. Its reliability and validity have been confirmed in Chinese research [27,52]. The PTGI comprises 21 items in five dimensions: new possibilities (e.g., “I’m more likely to try to change things that need changing”; five items), relating to others (e.g., “Knowing that I can count on people in times of trouble”; seven items), personal strength (e.g., “I believe I can be self-reliant”; four items), spiritual change (e.g., “I have a better understanding of spiritual support”; two items), and appreciation of life (e.g., “I would give priority to what is important in life”; three items). Teachers responded to these items on a 5-point Likert scale from 1 (strongly disagree) to 5 (strongly agree). The mean of the five dimension scores was the level of post-traumatic growth. The higher the score, the better the post-traumatic growth of teachers. In our study, the overall Cronbach’s α for the PTGI was 0.93, and the Cronbach’s α values for the new possibilities, relating to others, personal strength, spiritual change, and appreciation of life subscales were 0.93, 0.94, 0.96, 0.96, and 0.91, respectively.

Risk Perception. The measure used to assess risk perception was adapted from an index of questions about risk perception used in a previous study [22]. This questionnaire comprised eight items (e.g., “I’m in great danger of catching the virus”). Preschool teachers responded to the items on a 5-point Likert scale from 1 (strongly disagree) to 5 (strongly agree), with higher scores suggesting more severe risk perception. In our study, the Cronbach’s α for the risk perception index was 0.68.

Perceived Crisis Management. The measure used to assess perceived crisis management was adapted from an index of questions about crisis management during COVID-19 [22]. This assessment contained three items (e.g., “I think the government’s measures for COVID-19 prevention are effective”). Teachers responded to the items on a 5-point Likert scale from 1 (strongly disagree) to 5 (strongly agree). Higher scores corresponded with greater perceived crisis management. In our study, the Cronbach’s α for perceived crisis management was 0.96.

### 3.3. Procedure

We adapted preexisting questionnaires that collected information on preschool teachers’ post-traumatic growth, risk perception, and perceived crisis management. The risk perception and perceived crisis management tools were originally developed in English. We used the following measures to ensure the accuracy and readability of our translation. First, two doctoral students majoring in preschool education completed the translation separately. Second, we compared the two translations and discussed the differences, and reached consensus to form a draft. Third, experts in preschool education and mental health education, including a university faculty, a kindergarten manager, and an education expert, were consulted on the appropriateness and accuracy of the item wording. Finally, we revised the draft according to this feedback.

The preschool teachers in this study worked in preschools that enrolled children aged 3–6 years in China. In the formal stage of data collection, we recruited participants from the National Training Program for Preschool Teachers during the COVID-19 epidemic in 2020 in China. The preschool teachers provided consent to participate in this study after receiving information about the research objectives. Participants were assured that the information collected would be used solely for research purposes. We distributed electronic questionnaires (e-questionnaires) to participating preschool teachers from September to December 2020 using Wenjuanxing, a professional online questionnaire collection platform.

To ensure the objectivity of the data, we defined clear submission principles: the same participant and questionnaires from the same IP address could not submit the e-questionnaire repeatedly. To ensure participants answered questions openly and honestly, the e-questionnaire was sent directly to participants via anonymous links. The research was carried out in accordance with ethical standards for the treatment of human participants, and the study was approved by the Institutional Review Board at the study’s home institution.

### 3.4. Data Analysis

**Figure 1:**
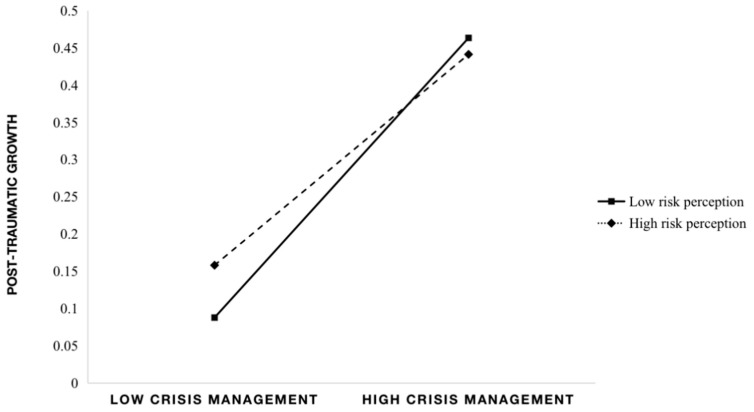
Moderating role of risk perception in the relationship between perceived crisis management and post-traumatic growth (*n* = 2921). Note. Values of the variables were centralized.

We used IBM SPSS version 26.0 (International Business Machines Corporation, Armonk, NY, USA) for the statistical analyses. First, we divided the classification of COVID-19 risk levels into low-risk, medium-risk, and high-risk based on previous research [53,54]. Data for confirmed cases in participants’ areas of residence were based on information published by the National Health Commission of the People’s Republic of China from 28–31 December 2020. Second, we examined the descriptive statistics for teachers’ post-traumatic growth, risk perception, and perceived crisis management. To explore the relationships among these factors, we conducted a partial correlation analysis after controlling for sex, age, educational background, years of teaching experience, type of preschool, and COVID-19 risk level. Third, we conducted a one-way analysis of variance (ANOVA) to clarify the difference in post-traumatic growth among preschool teachers living in different areas with different COVID-19 risk levels. Finally, we tested the moderating role of risk perception through the SPSS process plug-in (moderating effect model shown in Figure 1). Perceived crisis management was the independent variable (X), post-traumatic growth was the dependent variable (Y), the moderating variable was risk perception (M), and teachers’ sex, age, educational background, years of teaching experience, type of preschool, and area risk level were used as control variables. According to the statistic principles [55], the values for post-traumatic growth, risk perception, and perceived crisis management in this study were centralized separately.

## 4. Results

### 4.1. Profile of Post-Traumatic Growth among Preschool Teachers

The descriptive statistics and bivariate correlations for the key study variables are shown in Table 2. The mean score for post-traumatic growth was 3.56, indicating that preschool teachers’ post-traumatic growth was at an intermediate level in the context of COVID-19. Specifically, the mean ± standard deviation (SD) scores for the post-traumatic growth subscales (from high to low) were: appreciation of life (3.72 ± 0.98), spiritual change (3.59 ± 0.97), personal strength (3.57 ± 0.95), relating to others (3.56 ± 0.88), and new possibilities (3.38 ± 0.91). The mean score for risk perception was 3.86, indicating that preschool teachers’ risk perception was above a medium level during COVID-19. The mean score for perceived crisis management was 4.02, indicating that preschool teachers’ perceived crisis management was at relatively high level. There was a weak relationship between perceived crisis management and post-traumatic growth (r = 0.05, *p* < 0.05), indicating that preschool teachers’ perceived crisis management may have had a potential impact on post-traumatic growth. However, the exact relationships among these variables needed to be further explained by the moderation analysis.

### 4.2. Differences in Post-Traumatic Growth, Risk Perception, and Perceived Crisis Management

The results of the one-way ANOVA are shown in Table 3. There was no significant difference in post-traumatic growth among preschool teachers in different risk level areas, but there were significant differences in risk perception and perceived crisis management in different risk level areas. It should be noted that the effects of risk registration on risk perception and perceived crisis management were limited (η^2^*_partial_* < 0.2). The multiple comparative analysis showed that in high- and medium-risk areas, preschool teachers’ risk perception was significantly higher than that among teachers in low-risk areas. Furthermore, teachers’ risk perception in high-risk areas was significantly higher than that among teachers in medium-risk areas. Teachers’ perceived crisis management in high-risk areas was significantly higher than that among teachers in low-risk areas, and perceived crisis management in medium-risk areas was marginally higher than that among teachers in low-risk areas. However, teachers’ perceived crisis management in high-risk areas did not significantly differ from that among teachers in medium-risk areas.

### 4.3. Moderating Role of Risk Perception

The results of the moderation analysis are shown in Table 4. The interaction between risk perception and crisis management perception was significant after controlling for other variables (B = −0.046, 95% confidence interval [CI]: −0.088, −0.005; *p* < 0.05). Risk perception significantly moderated the relationship between perceived crisis management and post-traumatic growth (ΔR^2^ = 0.002, F [1,2163] = 4.7283; *p* < 0.05). We conducted a simple slope test to further explore the moderating role of risk perception in the relationship between crisis management and post-traumatic growth. There was a positive relationship between post-traumatic growth and perceived crisis management when risk perception was lower than one SD of the mean (B = 0.072, standard error (SE) = 0.032, 95% CI: 0.008, 0.135; *p* < 0.05). No relationship was found between post-traumatic growth and perceived crisis management when risk perception was higher than one SD of the mean (B = −0.021, SE = 0.027, 95% CI: −0.075, 0.032; *p* > 0.05) (See Figure 1).

## 5. Discussion

In this study, we found that preschool teachers’ post-traumatic growth in the context of COVID-19 was at an intermediate level, and there was no difference in different risk areas. However, preschool teachers’ post-traumatic growth was significantly related to risk perception and perceived crisis management, and risk perception had a significant moderating effect on the relationship between perceived crisis management and post-traumatic growth.

### 5.1. Differences in Post-Traumatic Growth in Different Risk Areas

We found that preschool teachers’ post-traumatic growth during COVID-19 was at an intermediate level, and “appreciation of life” had the highest score among all subscales. These results were consistent with a previous study [19]. Our results suggested that preschool teachers experienced a moderate positive psychological change during COVID-19. The change in “appreciation of life” was most obvious, which indicated that they were able to prioritize what was important in life, appreciate their life value, and enjoy every day after experiencing COVID-19. These results confirmed that the disaster triggered positive psychological changes. Moreover, we found that there was no significant difference in post-traumatic growth among preschool teachers living in areas with different COVID-19 risk levels. Although the Chinese government implemented different management strategies for different areas based on the level of COVID-19 risk, its serious attitude toward reducing pandemic risk was similar across all areas, which resulted in preschool teachers in different risk areas feeling sufficient support irrespective of level of risk (high-, medium-, or low-risk). Therefore, the severity of COVID-19 risk did not have a significant impact on post-traumatic growth. In addition, a previous study found the severity of a traumatic event had no direct impact on post-traumatic growth, and the effect of the trauma severity on post-traumatic growth depended on the person’s cognitive assessment of the event [43]. Our results suggested that further exploration of this issue is necessary.

### 5.2. Post-Traumatic Growth and Perceived Crisis Management

Our study found a positive correlation between perceived crisis management and post-traumatic growth among preschool teachers. Previous studies showed that social support was positively related to post-traumatic growth [24]. Our results of this study confirmed that the government’s crisis management, which represented a special type of social support, promoted post-traumatic growth in the context of COVID-19. Government crisis management affects public sentiment [41,42]. During COVID-19, some researchers found that the government’s positive actions to fight the virus alleviated negative emotions when people were in a state of high fear and increased their trust in society [22,23,38]. In China, the government announced the suspension of public transportation and other public gatherings after the outbreak of COVID-19 to prevent the spread of infection. In hindsight, these measures were timely and effective. International health organizations such as the World Health Organization expressed appreciation for the efforts of the Chinese government in the fight against COVID-19. In addition, the Chinese government protected the wages and benefits of preschool teachers during the COVID-19 pandemic [57,58]. These effective measures meant that preschool teachers perceived sufficient crisis management, which reduced their worries and negative emotions and may have promoted post-traumatic growth.

### 5.3. Post-Traumatic Growth and Risk Perception

We found a positive correlation between post-traumatic growth and risk perception among preschool teachers. This was consistent with the results reported by Calhoun et al. (2000) and Yang et al. (2021) [19,49], and suggested that deliberate cognitive processing is crucial to growth outcomes. Traumatic events tend to affect individuals’ basic cognitive schema, which triggers painful emotions and confusion. Faced with this challenge, people must reflect on their negative emotions repeatedly to alleviate these emotions [15]. COVID-19 is a public health emergency with major risks. Although the fatality rate was not high in China, its severity and infectivity and many other unknown aspects created a sense of crisis and threat among preschool teachers. Under such circumstances, teachers recognized the threat of COVID-19 to avoid infection. In addition, media such as television and newspapers improved people’s risk perception [59]. Media coverage of COVID-19, including the number of people diagnosed and preventive methods, increased preschool teachers’ scientific perceptions of COVID-19. Furthermore, the fact that the Chinese government effectively controlled COVID-19 meant that preschool teachers realized it was controllable. These cognitive factors helped preschool teachers to better understand the virus, avoid blind pessimism, and reduced “blind panic,” which meant they gained a certain degree of post-traumatic growth.

### 5.4. Moderating Role of Risk Perception

We found that risk perception moderated the relationship between perceived crisis management and post-traumatic growth among preschool teachers. Perceived crisis management had a significant positive impact on post-traumatic growth when the level of risk perception was appropriate. However, it had no significant impact on post-traumatic growth when the level of risk perception was high. This confirmed that post-traumatic growth is a cognitive process, and individual cognitive factors are more critical than external factors [15,27]. Post-traumatic growth is the result of an individual’s own efforts through the process of cognitive processing [25]. Therefore, compared with governmental crisis management, risk perception (which is part of an individual’s cognition) had a greater impact on post-traumatic growth. When risk perception was at an appropriate level, the emotional state of preschool teachers was relatively stable, and they could make a rational analysis of the COVID-19 crisis. In this condition, teachers perceived external support and protection, meaning the crisis management effectively promoted their post-traumatic growth. However, when risk perception was high, preschool teachers were prone to panic, and even suffered from mental health problems such as anxiety and depression. In this condition, negative emotions were dominant, and teachers had difficulty analyzing the crisis and achieving objective perceptions of COVID-19. Therefore, crisis management, as a type of external support, could not play a major role in forming post-traumatic growth.

## 6. Conclusions

This study explored a basic profile of post-traumatic growth among preschool teachers during COVID-19 and the moderating role of risk perception in the relationship between perceived crisis management and post-traumatic growth. This study found that preschool teachers in China had an intermediate level of post-traumatic growth, and there was no significant difference in post-traumatic growth by risk level area. Post-traumatic growth was significantly related to risk perception and perceived crisis management, and risk perception moderated the relationship between perceived crisis management and post-traumatic growth. These results contribute to developing targeted practical measures to improve preschool teachers’ mental health in the context of COVID-19.

## 7. Implications for Practice

This study explored the relationships among post-traumatic growth, risk perception, and perceived crisis management in preschool teachers during COVID-19 and confirmed the moderating role of risk perception. These findings deepened our perspectives on the mechanism of preschool teachers’ post-traumatic growth and potential strategies for improving their mental health during a public health emergency. We put forward the following suggestions based on our results. First, local governments and preschool managers should pay attention to preschool teachers’ post-traumatic growth. This not only affects the mental health of teachers but also has an important effect on children’s growth [6,12,30]. Second, state and local governments should play a key role in crisis management during COVID-19, as teachers perceive such external support and may therefore engage in more rumination [22]. Furthermore, some appropriate and targeted measures should be implemented, such as providing thematic training on COVID-19 for preschool teachers, decreasing their work burden, and setting up risk insurance funds [9,60,61]. In particular, state and local governments should strengthen humanistic care and provide psychological intervention assistance to preschool teachers in need in the context of COVID-19.

## 8. Limitations

This study had several limitations. First, our findings were obtained from cross-sectional data. The occurrence of post-traumatic growth takes a certain amount of time, and its positive effect is achieved over time [62], whereas this study only collected cross-sectional data about post-traumatic growth. Second, the Cronbach’s α for the risk perception index was somewhat unsatisfactory. Finally, this study only investigated the impact of crisis management and risk perceptions on post-traumatic growth, and other contributing factors were not explored. A further study is needed to track changes in post-traumatic growth among preschool teachers in the context of COVID-19 [63]. We also need to revise and improve the risk perception scale used in this study to increase adaptability in China. In addition, we need to measure teachers’ post-traumatic growth to use questionnaires, interviews, and observations comprehensively. Finally, it is necessary to explore the impact of other factors on preschool teachers’ post-traumatic growth and verify methods for improving their post-traumatic growth through an intervention study.

## Figures and Tables

**Table 1:** Sociodemographic characteristics of survey participants (*n* = 2921).

Variables	Categories of Variable	%
Teacher Sex	Male	3.5
	Female	96.5
Teacher Age	Younger than 30 years old	32.1
	31–35 years old	23.9
	36–45 years old	19.2
	Older than 46 years old	24.8
Educational background	Senior high school or below	13.7
	Up to 3-year college education	47.4
	4-or-more-year university education or higher	36.9
Years of teaching experience	Less than 3 years	34.7
	3–5 years	24.5
	6 years or above	40.8
Type of preschool	Public	66.3
	Private	33.7
COVID-19 risk level of area	Low-risk area	23.1
	Medium-risk area	41.5
	High-risk area	35.4

**Table 2:** Correlations and descriptive statistics for the main study variables (*n* = 2921).

Variables	1	2	3
1. Post traumatic growth	-		
2. Risk perception	0.20 ***	-	
3. Perceived crisis management	0.05 *	0.14 ***	-
Mean	3.56	3.86	4.02
Standard deviation	0.84	0.57	1.16

Note. * *p* < 0.05, *** *p* < 0.001 (two-tailed).

**Table 3:** Differences in variables by different risk level areas (*n* = 2921).

Variables	Low risk Area	Medium Risk Area	High Risk Area
Post-traumatic growth	3.54 ± 0.81	3.77 ± 0.58	3.91 ± 1.14
Risk perception	3.56 ± 0.94	3.85 ± 0.57	4.04 ± 1.18
Perceived Crisis management	3.57 ± 0.73	3.94 ± 0.55	4.06 ± 1.02
F	0.228 ^n.s.^	18.174 ***	3.679 ***
η^2^*_partial_*	-	0.013	0.003

Note. *** *p* < 0.001, n.s. = non-significant with *p* > 0.05 (two-tailed), η^2^*_partial_* < 0.2, indicating a small effect [56].

**Table 4:** Results of the moderator analysis (*n* = 2921).

Variables	B	SE B	t	95% CI
Constant	0.276	0.248	1.113	[−0.209, 0.761]
W: risk perception	0.188	0.021	9.115 ***	[0.147, 0.228]
X: perceived crisis management	0.024	0.021	1.167	[−0.016, 0.064]
X × W	−0.046	0.021	−2.174 *	[−0.088, −0.005]
C_1_ risk level	0.009	0.027	0.347	[−0.044, 0.063]
C_2_ gender	−1.000	0.116	−0.866	[−0.328, 0.127]
C_3_ educational background	0.041	0.032	1.260	[−0.023, 0.104]
C_4_ age	−0.009	0.022	−0.438	[−0.053, 0.033]
C_5_ nature of kindergarten	−0.152	0.045	−3.421 ***	[−0.239, −0.065]
C_6_ working experience	0.025	0.029	0.832	[−0.033, 0.082]

Note. * *p* < 0.05, *** *p* < 0.001 (two-tailed), C_1_–C_6_: control variables.

## Data Availability

Not applicable.
